# Hemicellulosic Polysaccharides From Bamboo Leaves Promoted by Phosphotungstic Acids and Its Attenuation of Oxidative Stress in HepG2 Cells

**DOI:** 10.3389/fnut.2022.917432

**Published:** 2022-06-13

**Authors:** Zhuqian Xiao, Jiajie Li, Hongpeng Wang, Qiang Zhang, Qing Ge, Jianwei Mao, Ruyi Sha

**Affiliations:** ^1^Zhejiang Provincial Collaborative Innovation Center of Agricultural Biological Resources Biochemical Manufacturing, Zhejiang University of Science and Technology, Hangzhou, China; ^2^College of Chemical Engineering, Zhejiang University of Technology, Hangzhou, China

**Keywords:** bamboo leaves, hetero-polysaccharides, oxidative stress, HepG2 cells, phosphotungstic acid

## Abstract

In this work, we exploited an efficient method to release hemicellulosic polysaccharides (BLHP) from bamboo (*Phyllostachys pubescens Mazel*) leaves assisted by a small amount of phosphotungstic acid. Structural unit analysis proved that BLHP-A1 and BLHP-B1 samples possessed abundant low-branch chains in →4)-β-_D_-Xyl*p*-(1→ skeleton mainly consisting of Xyl*p*, Man*p*, Glc*p*, Gal*p*, and Ara*f* residues. According to the results of the antioxidant activity assays *in vitro*, both of the two fractions demonstrated the activity for scavenging DPPH⋅ and ABTS^+^ radicals and exhibited relatively a high reducing ability compared to the recently reported polysaccharides. Moreover, the antioxidant activities of purified polysaccharides were evaluated against H_2_O_2_-induced oxidative stress damage in HepG2 cells. BLHP-B1 showed more activity for preventing damages from H_2_O_2_ in HepG2 cells by improving the enzyme activities of SOD, CAT, and GSH-Px and decreasing the production of MDA as well as suppressing reactive oxygen species (ROS) formation. This study implied that BLHP could demonstrate its attenuation ability for oxidative stress in HepG2 cells.

## Introduction

Free radicals [reactive oxygen species (ROS) and nitrogen species are included] act as essential active species in living tissues to maintain cellular homeostasis in organisms. The accumulation of radicals can lead to some serious diseases including Alzheimer’s disease, Parkinson’s disease, and even tumors (1–3). On the other hand, the activity of the antioxidant enzyme system, including catalase (CAT), superoxide dismutase (SOD), and glutathione peroxidase (GSH-Px), will be motivated to scavenge the active radicals, protecting cells from the invasion of ROS. Oxidative stress from ROS has been widely regarded as a significant factor in cell aging and immune injuries, and H_2_O_2_ is one of the general contributors to cause oxidative damage in model cells or animal tissue evaluation (4, 5). Recently, quite a few natural products from terrestrial and aquatic organisms, including peptides, glycoproteins, terpenoids, and polysaccharides, have been elucidated to possess promising antioxidant activity due to a particular structure (6–8). Polysaccharides, a polymer widely found in organisms, are rich in hydroxyl groups and functional side chains, exhibiting excellent antioxidant activity (9). A quintessential example should be cited that distinct antioxidant, antitumor, and antimicrobial activities of polysaccharides extracted from Ganoderma have been reported previously (10). The type of glycosidic linkages, branching patterns, and linkages to proteins in Ganoderma polysaccharides are all concerning its antioxidant activity.

Bamboo, one of the agricultural and forestry cash crops, is widely cultivated in southeast Asia. Through the ages, the bamboo stem has been used as a resource for paper manufacturing due to its 40–48% of cellulose (11). Likewise, the contents of cellulose and hemicellulose in bamboo leaves are 20–40% and 35–45%, respectively (12). Particularly, the content of hemicellulose is more in the bamboo stem, which is suggestive of the occurrence of structural carbohydrate polymers with various branches. Bamboos encompass 1,250 species within 75 genera and share the desirable characteristics of high productivity and are fast-growing, and could be recognized as renewable resources to extract functional hetero-polysaccharides because of their abundant hemicelluloses (13). However, the extraction methods of plant-based polysaccharides were adopted as a physical process assisted by ultrasonic (14), microwave (15), and even hot water (16) directly, which proved to show low selectivity for specific components releasing. The crude hemicelluloses extracted from plant sources probably contain _D_-galactose, _D_-mannose, _L_-rhamnose, _L_-fucose, and even peptides, implying that it is a challenge to analyze the characteristic structure of polysaccharides after extraction using different physical methods. Phosphotungstic acid (HPW, H_3_PW_12_O_40_), one of the heteropolyacids with Keggin structure, is a strong Brønsted acid. According to our previous study, a certain amount of HPW demonstrated high selectivity for xylose recovery (17). Hence, it is reasonable to apply phosphotungstic acid in the selective extraction of hemicellulosic polysaccharides. On the other hand, numerous publications elaborate that multitudinous polysaccharides extracted from plants exhibit strong free radicals scavenging ability as well as an excellent protective effect on oxidative damage of ROS in induced cells *in vitro* (18–20). Moreover, cancer cells with rapid proliferation capacity demonstrate a high utilization rate of inspired oxygen. It is believed that these cells are subjected to the influence of oxidative stress. Coupled with their easy culturing, we plan to employ H_2_O_2_-induced HepG2 cells damage *in vitro* as a model to evaluate their protective effects on the oxidative injury. The structure of the extracted polysaccharides samples will also be analyzed.

## Materials and Methods

### Materials and Chemicals

One-year-old bamboo (*Phyllostachys pubescens Mazel*) leaves were collected from a mountain in Xihu District, Hangzhou, Zhejiang Province of China. All the raw materials were smashed into about 200 μm (all the particle sizes were measured on a Topsizer Plus laser particle size analyzer, Zhuhai OMEC Instruments Co., Ltd., Zhuhai, China) after oven drying. Dimethyl sulfoxide (DMSO), 1,1-diphenyl-2-picrylhydrazyl radical (DPPH), 2,2-azinobis-3 ethyl benzothiazoline-6-sulphonic acid (ABTS), standard monosaccharides including _D_-glucose, _D_-galactose, _L_-arabinose, _L_-rhamnose and _D_-mannose, potassium ferricyanide were purchased from Sigma-Aldrich in China mainland. H_2_O_2_, FeSO_4_, and phosphotungstic acid were from Sinopharm Chemical Reagent Co. Ltd., China. DEAE-Sepharose^®^ fast flow column (2.0 cm × 40 cm, Cl^–^ form) is purchased from Sigma-Aldrich, Shanghai, China. Commercial assay kits for the determination of SOD, catalase (CAT), GSH-Px, and methylenedioxyamphetamine, (MDA) including Cell Counting Kit-8 (CCK-8), and BCA Protein Assay Kit, Radio Immunoprecipitation Assay (RIPA) lysis buffer, and Minimum Eagle’s medium (MEM) were purchased from Beyotime Biotechnology Co. Ltd., Shanghai, China. Reactive Oxygen Species Assay Kit (DCFH-DA) was from MedChemExpress (Shanghai).

### Bamboo Leaves Hetero-Polysaccharides Extraction and Purification

Bamboo leaves powder (BLP) was further ball-milled for 3.0 h to obtain tinnier particles in nearly 50 μm diameter (particle size analyzer measured). The milled powder was then dewaxed with 95% ethanol at 60°C before extraction. To measure the yield of polysaccharides, the dewaxed BLP was oven-dried till the moisture was nearly 4.0%. In a typical extracting process, briefly, 5.0 g BLP mixed with 0.1 g phosphotungstic acid was employed in the Teflon-lined ultrasonic reactor (100 ml, Yanzheng Instrument Co., Ltd., China) at 50 W and 60.0 ml deionized water was injected as the extracting medium. This weak hydrolysis occurred at 80°C for 2.0 h under N_2_ atmosphere. After extraction, the liquid was filtrated, and the trace phosphotungstic acid was removed by dialysis (3,000 molecular cutoff by regenerated cellulose membrane from CMEC Biochemical Co. Ltd., China). The neutral filtrate was then concentrated by 95% alcohol precipitation and then centrifuged at 8,000 rpm for 20 min to yield crude bamboo leaves polysaccharides (CBLP). Successively, CBLP precipitates were freeze-dried to obtain CBLP powder. DEAE-Sepharose fast-flow column (2.0 cm × 40 cm, Cl^–^ form) was applied to the isolation of components. The concentrated NaCl solutions (0.1, 0.2, 0.4, 0.6, 1.0, and 2.0 mol/L) were added as the eluent at the rate of 1.0 ml/min after 500.0 ml deionized water elution. The concentration of isolated polysaccharides was measured according to the absorbance at 498 nm by the phenol-sulfuric acid method (21, 22). The tubes containing target components were then collected and evaporated to concentrate the polysaccharides solutions accordingly. The samples were further purified by the DEAE Sephacryl S-300 column to obtain purified bamboo leaves hetero-polysaccharides (BLHP). Finally, these concentrated solutions were freeze-dried at –60°C (nominated as BLHP-A1 and BLHP-B1).

### Chemical Analysis and Physical Characterizations of Bamboo Leaves Hetero-Polysaccharides

The monosaccharides were detected by high-performance anion-exchange chromatography with pulsed amperometric detection (HPAEC-PAD, Dionex ICS-5000^+^, Thermo Fisher Scientific, United States). The hydrolysate was determined by an ED detector with a picomole level resolution. The analytical column was Carbo PAC™ PA10 (4 mm × 250 mm) protected by PAC™ PA10 (4 mm × 50 mm) column. The chromatographic pure sugars, including _D_-glucose, _D_-galactose, _L_-arabinose, _L_-rhamnose, _D_-mannose, and the hydrolysate samples were injected into the detector with 200 mM NaOH eluent at 1.0 ml/min for 40 min. The column temperature was 40°C.

#### Fourier Transform Infrared Spectrometer

The characteristic covalent bonds and groups of BLHP fractions were analyzed on a Bruker V70 IR Spectrometer (Bruker, Germany) in 4,000–400 cm^–1^. Before the performance, trace quantities of the sample were grounded with spectrographic grade potassium bromide and then pressed into a pellet under 12.0 MPa pressure.

#### Ultraviolet Analysis

The polysaccharides (1.0 mg) were dissolved into 1.0 ml ultrapure water, and then scanned and analyzed on a Ultraviolet (UV)-vis spectrophotometer (UV-5500PC, Metash Instruments Co. Ltd., Shanghai, China) within the wavelength range of 200–400 nm.

#### Scanning Electron Microscopy Characterization

To observe the morphologies of polysaccharides after freeze-drying, Scanning Electron Microscopy (SEM) analysis was performed on a HITACHI S 4800 SEM (HITACHI, Japan) with an accelerating voltage of 15.0 kV. The samples were coated with gold atoms to strengthen the conductivity.

#### Methylation Analysis

First, polysaccharides samples were vacuum dried for 24 h in a dryer with P_2_O_5_; 10.0 mg of sample was dissolved in 6.0 ml of DMSO and stirred for 15 h at 45°C. Then 30.0 mg NaOH was added to the solution above and stirred for 4.0 h at room temperature. Successively, 3.6 ml of methyl iodide was added into the solution under nitrogen protection in a dark place. After stirring for 1.0 h at 25°C, 6.0 ml of deionized water was introduced to terminate the reaction. The obtained mixture was extracted by trichloromethane and the water phase was removed. The residual organic phase was further extracted by deionized water five times. Then the organic phase was distilled to obtain methylated samples. This methylation procedure was repeated three times to ensure complete methylation. Afterward, the methylated samples were hydrolyzed with 2 M of TFA (2 ml) for 2 h at 120°C, and then reduced by NaBD4 (60 mg) overnight at room temperature. The dry reduced samples were then acetylated by acetic anhydride (0.5 ml) in pyridine (0.5 ml) at 100°C for 2 h to gain their partially methylated alditol acetates, which were analyzed by gas chromatography-mass spectrometry (GC–MS).

#### Gas Chromatography-Mass Spectrometry Analysis

The methylated alditol acetates were analyzed on an Agilent 7890A-5975C (Agilent Technology, Palo Alto, CA, United States) with an Rtx-5 quartz capillary column (0.25 mm × 0.25 mm × 30 m, Shimadzu Technology, Kyoto, Japan). The derived sample (1.0 μl) was injected with a He flow of 1.0 ml/min. The temperature program was set as follows: (1) initially 120°C for 5.0 min and then to 200°C at 5°C/min; (2) continuously increased to 215°C/min at 2°C/min; (3) increased to 270°C at 20°C/min and then maintained for 5 min. Mass spectra were recorded at a range of 40–500 m/z.

#### NMR Analysis

A total of 20.0 mg of polysaccharides powder was dissolved into 0.5 ml HDO in an NMR tube. The filtrates were recorded on a 600 MHz Digital spectrometer (AVANCE III 600 MHz, Bruker Corporation, New Castle, DE, United States) to obtain ^1^H and ^13^C spectra, and two-dimensional (2D) NMR spectra (COSY, HSQC, and HMBC).

### The Chemical Antioxidant Activity Evaluation *in vitro* of Bamboo Leaves Hetero-Polysaccharides

#### DPPH⋅ and ABTS^+^ Free Radicals Scavenging

DPPH⋅ free radical scavenging tests were performed according to the standard DPPH⋅ assay method developed by Jiang (23) with a slight modification. A total of 4.0 ml of DPPH⋅ solution (0.1 mM in 95% MeOH) was mixed with a specific volume of concentrated BLHP filtrate. A total of 45 μl of Tris-HCl buffer (450 mM, pH = 7.4) was injected into the mixture and then incubated in the dark for 30 min at 30°C after being vibrated well. The absorbance of the liquid was read at 517 nm. The radical scavenging activity was then calculated by Eq. (1):


$$DPPHscavengingrate\left(\%\right)=\left[1-\left(A_{1}-A_{2}\right)/A_{0}\right]\times 100\%$$

The ABTS^+^ radical scavenging of BLHP was evaluated using the method developed by Ma (24).

#### Reducing Ability Test

The reducing ability of BLHP was measured according to our previous method (25) with minor modification. Briefly, 1.0 ml of concentrated BLHP solution was mixed with 2.5 ml of 0.2 M phosphate buffer (pH = 6.6) and 2.0 ml of K_3_Fe(CN)_6_ solution (1%, w/v). The mixture was centrifuged for 10 min after reacting for 20 min in a 50°C water bath. A total of 2.0 ml of supernatant and 2.0 ml of deionized water were then mixed before 0.4 ml FeCl_3_ solution (1%, w/v) was added. This solution was stewed for 10 min at 25°C. Finally, the absorbance was recorded at 700 nm, and vitamin C (VC) was used as the positive control.

### Bamboo Leaves Hetero-Polysaccharides Attenuation in Oxidative Stress Induced by H_2_O_2_ in HepG2 Cells

#### Cell Lines and Culture Conditions

The human hepatocellular carcinomas (HepG2, CL-0103) cell line was purchased from Procell Life Science and Technology Co. Ltd. (Wuhan, China), authenticated by Short Tandem Repeat (STR). After thawing, the cells were cultured in minimum Eagle’s medium (MEM) with 10% FBS, 1% non-essential amino acids (NEAA), and 1 mM sodium pyruvate (NaP) and then incubated at 37°C under a 5% CO_2_ atmosphere. In this work, HepG2 cells were cultured for the measurement of the protective effect on H_2_O_2_-induced oxidative damage. The differentiation method was conducted according to previous studies (26). Cells were subcultured every 5 days by trypsinization with 0.05% trypsin-EDTA solution.

#### Cytotoxicity Assay

The toxicity of polysaccharides samples (BLHP-A1 and BLHP-B1) toward natural HepG2 cells was evaluated by the MTT method (27); 5 × 10^5^ cells/well were plated in a flat-bottom (96-well microtiter plate) and incubated under the culturing condition for 24 h. Then the cells were treated with appropriate solutions of BLHP-A1 and BLHP-B1 (250, 500, 750, 1,000, 2,000, and 3,000 μg/ml) and incubated for 24 h at 37°C under a 5% CO_2_ atmosphere. After that, the medium was replaced with a fresh medium and then 100 μl MTT (3-(4,5-dimethylthiazol-2)-2,5-diphenyltetrazolium bromide, 1.0 mg/ml in PBS buffer) was added (28). The plates were incubated for 1.0 h at 37°C. Successively, the medium was removed carefully and 200 μl DMSO (dimethyl sulfoxide) was introduced to dissolve the formazan crystals. After oscillating for 10 min in the dark, the absorbance at 570 nm was recorded in an ELISA instrument (SpectraMax iD3, Molecular Devices Co. Ltd., Shanghai, China).

#### H_2_O_2_-Induced Oxidative Damage of HepG2 Cells

Generally, H_2_O_2_ could react with metallic cations in low valence such as Fe^2+^ and Cu^+^, and some hydroxyl radicals generated during the reaction would lead to oxidative damage in cells. The HepG2 cells were cultured under the same condition described before. For a typical H_2_O_2_-induced cell oxidative damage assay, briefly, the different concentrations (100–1,300 μmol/ml) of H_2_O_2_ were employed to treat the cultured HepG2 cells for 4.0 h. The absorbance at 540 nm was determined. MTT was introduced to each well. The blank group was treated with MEM in the absence of H_2_O_2_.

#### Reactive Oxygen Species Measurement

The intracellular ROS were measured by ROS assay kit with a modification of Ma’s method (29). The cells in the logarithmic phase were inoculated in a 96-well microtiter plat with 1 × 10^6^ cells/well and then incubated for 24 h at 37°C under a 5% CO_2_ atmosphere. After being treated with the low, medium, and high concentrations of BLHP, 10 μmol/L of DCFH-DA fluorescent probe was introduced to the cell suspension. The intensity of ROS was determined on flow cytometry (CytoFLEX, Beckman Coulter Co. Ltd., Brea, CA, United States) with the analysis software of CytExpert.

#### Protective Measurement of Bamboo Leaves Hetero-Polysaccharides on H_2_O_2_-Induced Oxidative Damage in HepG2 Cells

In the previous description, the model of H_2_O_2_-induced HepG2 cells oxidative stress was established. To explore the protective effect of BLHP on damaged cells, HepG2 cells were cultured under the conditions described in the “Cell Lines and Culture Conditions” and “Cytotoxicity Assay” sections. After incubation for 24 h, the evaluations were conducted, respectively, as follows: (1) The final setting concentrations of BLHP samples (250, 1,000, and 3,000 μg/ml) were employed in the protected groups. (2) The damage group was treated with 600 μmoL/ml H_2_O_2_ without any BLHP in MEM. (3) The cells in the control group (NC) were cultured in MEM and then treated without any other agents. After 48 h treatment with the BLHP samples, the protected groups and damage groups were challenged with 600 μmoL/ml H_2_O_2_ for another 4.0 h to induce oxidative stress and the control group was treated with MEM at the same condition. After lysing of cells, the system was centrifuged for 5 min at 12,000 *g* at 4°C to separate solids and liquid. The liquid supernatant was collected for the assays of enzyme activities and the measurement of some characteristic products in cells. In detail, the activities of SOD, CAT, and GSH-Px enzymes were evaluated. Moreover, since MDA was the indicator for lipid oxidation degree, from this view, the content of MDA was determined by assay kits.

#### Statistical Analysis

All the data were expressed as the means ± standard error of the mean (*n* = 3). *SPSS 2.0* was used for the statistical analysis of experimental data and the one-way ANOVA test was applied for the analysis of the statistically significant difference. A *P*-value of less than 0.05 was represented as significantly different.

## Results and Discussion

### Isolation and Purification of Bamboo Leave Hetero-Polysaccharides

**FIGURE 1:**
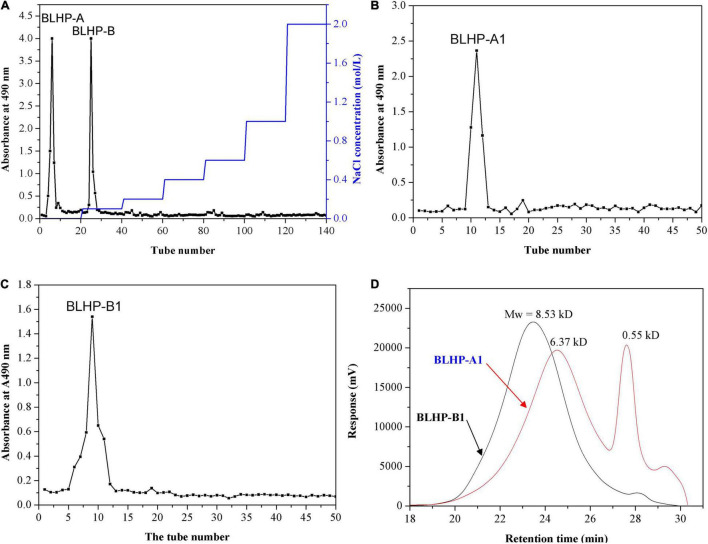
Purification of the BLHP samples: **(A)** the crude polysaccharides were applied to DEAE-Sepharose fast flow column (2.0 cm × 40 cm, Cl^–^ form) to produce BLHP-A and BLHP-B fractions; **(B)** DEAE Sephacryl S-300 column (2.0 cm × 40 cm) was applied to purify BLHP-A sample; **(C)** DEAE Sephacryl S-300 column (2.0 cm × 40 cm) was applied to purify BLHP-B sample; **(D)** chromatogram of the molar mass distribution of BLHP-A1 and BLHP-B1 detected by GPC.

The crude hemicellulosic polysaccharides from bamboo leaves were purified by a DEAE-Sepharose fast-flow column (2.0 cm × 40 cm, Cl^–^ form). According to absorbance statistics at 490 nm in Figure 1A, two fractions were obtained and denominated as BLHP-A and BLHP-B. The two fractions were from the elution of deionized water and 0.1 M NaCl solution, respectively. All assigned tubes were collected, concentrated, and freeze-dried to achieve BLHP-A and BLHP-B powder. Successively, the two samples were re-dissolved and further purified by DEAE Sephacryl S-300 column separately and the results are illustrated in Figures 1B,C. Interestingly, only one fraction was isolated from BLHP-A or BLHP-B despite BLHP-B1 showing wide molecular weight distribution in Figure 1D. This result indicated that BLHP-A and BLHP-B were homogeneous polysaccharides prepared by phosphotungstic acid hydrolysis. Moreover, according to gel permeation chromatography (GPC) analysis, the average molecular weights of BLHP-A1 and BLHP-B1 were 8.53 and 6.37 kDa, respectively. From these views, the addition of phosphotungstic acid during polysaccharides extraction will contribute significantly to controlling molecular weight to obtain polysaccharides in lower molecular weight (<10 kD). The trace phosphotungstic acid could be removed by dialysis easily. GPC analysis also exhibited two specific peaks in the BLHP-B fraction, probably implying the occurrence of oligosaccharides.

### Chemical Compositions and Physical Characterizations of Bamboo Leaves Hetero-Polysaccharides

**FIGURE 2:**
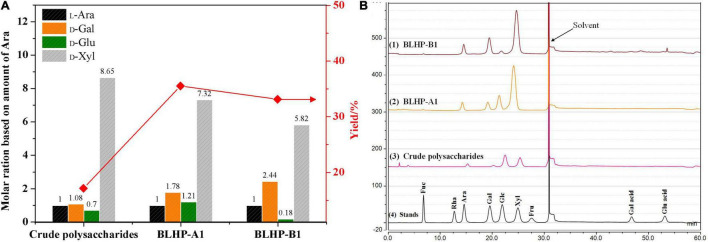
The monosaccharide content in crude polysaccharides, BLHP-A1 and BLHP-B1 samples. **(A)** The relative molar ratios of monosaccharides among three samples based on the molar of arabinose. **(B)** HPACE spectrum for monosaccharide detection. All the samples were prepared at concentrations of 1.0 mg/ml.

The monosaccharide compositions of BLHP-A1 and BLHP-B1 were detected quantitatively by HPAEC-PAD and the results are shown in Figure 2A. All the samples extracted from bamboo leaves seemingly demonstrated similar monosaccharide distributions, including _D_-glucose, _D_-xylose, _L_-arabinose, and _D_-galactose despite other trace monomers being detected in a specific sample. On the other hand, according to the high resolutions among monomers in Figure 2B, HPAEC could be an effective equipment for the separation of sugars. Especially, the relative contents of specific monosaccharides were different in samples. _D_-Xylose was the dominating component in all samples, implying our extracting method mainly facilitated the disassembly of hemicelluloses from bamboo leaves, which was the reason for hetero-polysaccharides definition in this work. Considering the content of xylose and similar distribution of monosaccharides, BLHP-A1 and BLHP-B1 could be defined as hemicellulosic polysaccharides, discriminating from cellulosic polysaccharides.

**FIGURE 3:**
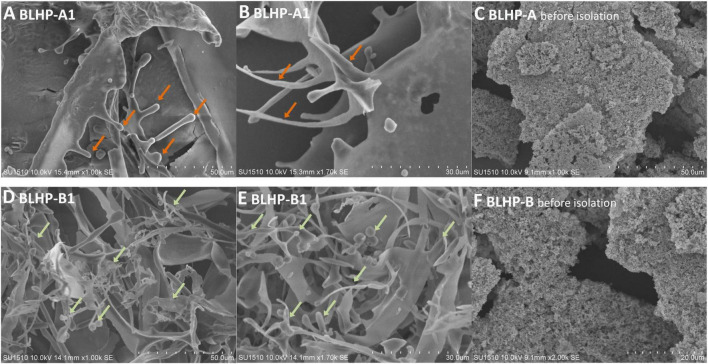
The morphologies of polysaccharides extracted from bamboo leaves. **(A,B)** Represent BLHP-A1 fraction after being purified by DEAE-Sepharose fast-flow column. **(D,E)** Illustrate purified BLHP-B1 fraction by DEAE-Sepharose fast-flow column. **(C,F)** Are the morphologies of BLHP before further purification.

The morphologies of BLHP are shown in Figure 3. The polysaccharides exhibited different surface structures before and after column purification. BLHP-A1 showed some irregular filaments at the margin of tiny flaps. BLHP-B1 analogously manifested a rough surface including sheet-like, rod-like, and lump-like structures. These parallel results were probably indicative of a filamentous structure formed by cross-linked chains, consistent with the structure of the purified polysaccharides extracted from auriculariales (30). It was universally acknowledged that the polysaccharides would recrystallize during freeze-dried and interpreted with inter- and intra-molecular hydrogen bonds. In contrast, polysaccharides exhibited uniform structures like granular aggregates and there was no significant discrepancy between BLHP-A and BLHP-B.

### Ultraviolet and Fourier Transform Infrared Analysis

**FIGURE 4:**
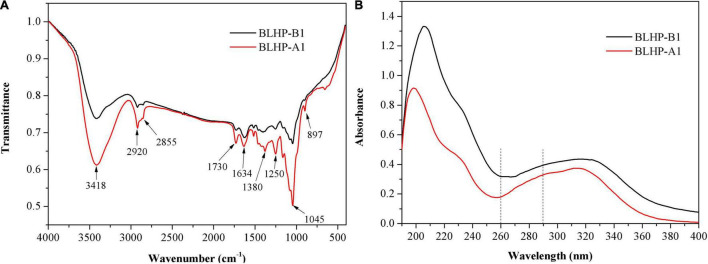
FT-IR spectra analysis **(A)** and UV full scanning **(B)** of BLHP-A1 and BLHP-B1 fractions.

Fourier transform infrared (FT-IR) analysis in Figure 4A demonstrated significant differences between BLHP-A1 and BLHP-B1 according to the infrared absorption. Concretely, both samples gave the characteristic absorbance at 3,418 cm^–1^ for O–H, 2,920 and 2,855 cm^–1^ for C–H (asymmetrical stretching) structures (31). These bonds were indispensable in polysaccharides. The absorbance at 1,730 cm^–1^ was assigned to the stretching vibration of carbonyl groups. The peak at 1,634 cm^–1^ was relevant to the flexural vibration of O–H (32) since no considerable content of protein was characterized in Figure 4B. The occurrence of a characteristic peak at 1,380 cm^–1^ might be attributed to trace amide (33). The bending vibration of O–H was observed at about 1,250 cm^–1^. The peak at 897 cm^–1^ was assigned to the stretching vibration of C–O–C and C–O–H, originating from pyranoid monosaccharides. On the other hand, the bands at 897 cm^–1^ were the characteristic absorption of β-configuration (22), which was consistent with NMR analysis. The absorbance at 1,045 cm^–1^ was suggestive of the presence of pyranose mainly from the cellulose fraction (34). The UV spectrum (Figure 4B) demonstrated no obvious absorbance peak at 260 and 290 nm approximately, which was indicative of the absence of free or combinative protein and nucleic acids in the two samples (35).

### Methylation Analysis

The glycosidic linkage types of BLHP-A1 and BLHP-B1 were explored by methylation analysis. The methylated products of the two samples were hydrolyzed and detected by GC–MS. As a result, there were five alditol acetates in BLHP-A1, including 2,3,4,6-Me_4_-Man*p*, 2,3,4,6-Me_4_-Glc*p*, 2,3-Me_2_-Xly*p*, 2,3,4,6-Me_4_-Gal*p*, 3,4,6-Me_3_-Man*p* and 2,4,6-Me_3_-Gal*p*. Comparatively, 2,3,4,6-Me_4_-Man*p*, 2,3,4,6-Me_4_-Glc*p*, 2,3-Me_2_-Xly*p*, 2,3,4,6-Me_4_-Gal*p*, 3,4,6-Me_3_-Man*p*, 2,3,4-Me_3_-Glc*p* and 2,3,4-Me_3_-Gal*p* derivative alditol acetates were detected in BLHP-B1. In addition, the linkage types of the two samples represented some differences; 1,6-linked-Gla*p* and 1,6)-linked-Glc*p* fragments were only present in BLHP-B1 while 1,3)-linked-Gal*p* was absent in BLHP-B1. However, the methylated alditol acetate about arabinose was absent in both polysaccharides despite a small quantity of arabinose being determined by HPAEC analysis probably due to incomplete methylation and isomerization during the derivatization process. Some extra groups including *O*-acetyl groups and terminal sugar needed confirmation by the NMR spectrum.

### NMR Analysis

**FIGURE 5:**
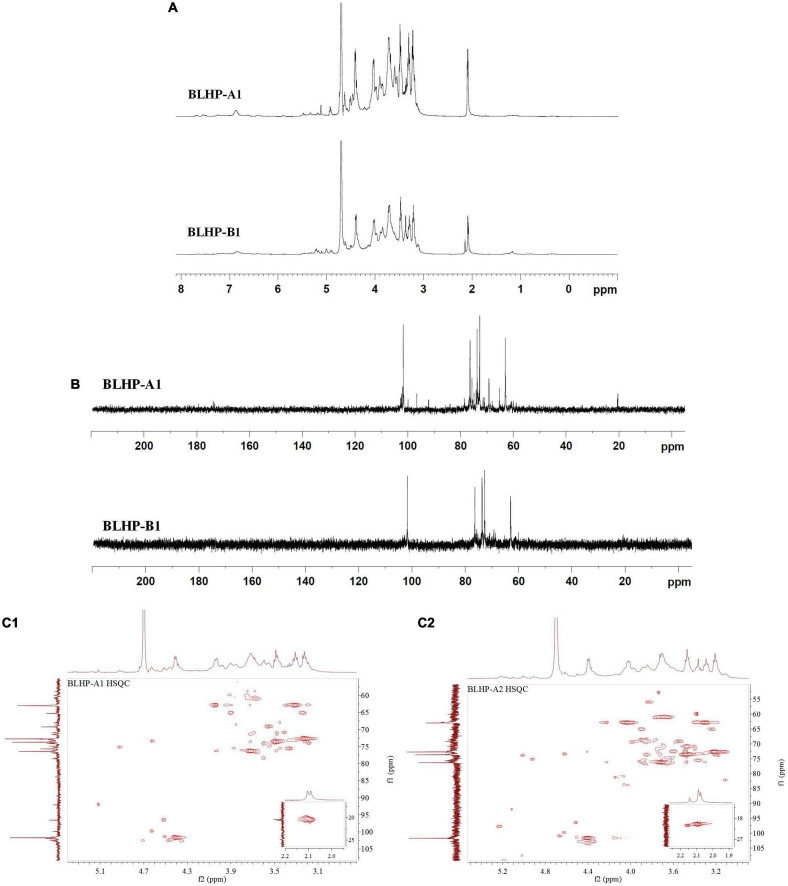
^1^H **(A)** and ^13^C **(B)** NMR spectra and HSQC for BLHP-A1 and BLHP-B1 samples **(C1,C2)** after deduction of some weak signal.

NMR analysis was the key technology in this work to provide sugar residue linkage sequence and anomeric configurations. ^1^H and ^13^C NMR spectra are shown in Figure 5. In the ^1^H-NMR spectra, lower field resonance in the chemical shift range of 1.9 to 6.0 ppm indicated the presence of polysaccharides (36, 37). The chemical shifts about anomeric protons in branched polysaccharides were observed from 4.3 to 5.5 ppm. The chemical shifts in protons correlating with C_2,3_ in sugar units varied from 3.2 to 4.2 ppm. Hence, the main monosaccharides were attributed to β-configuration since the signal peaks at 4.3–4.8 ppm demonstrated strong signals while the chemical shifts for α-configurations were near 5.0 ppm and lower chemical shifts. Notably, the main monosaccharide in both BLHP-A1 and BLHP-B1 was xylose according to the HPAEC analysis. Therefore, _D_-Xyl*p* linkage was attributed to β-configuration. According to ^1^H spectrum in Figure 5A, six anomeric proton signals (5.17, 4.81, 4.60, 4.52, 4.46, and 4.41 ppm) appeared in BLHP-A1. And seven anomeric proton signals (5.18, 5.03, 4.81, 4.60, 4.52, 4.48, and 4.42 ppm), were observed in BLHP-B1. The chemical shift at δ 4.70 ppm was HDO. In ^13^C spectrum in Figure 5B, the anomeric carbon signals were difficult to be determined because of the overlaps and trace amount. Still, we could also discriminate the main anomeric carbon signals, which delivered the structural information about hetero-branches in polysaccharides. The chemical shift at about 177 ppm was attributed to trace uronic acid in agreement with FT-IR analysis (38). The specific ^1^H and ^13^C cross assignments for chemical shifts of BLHP-A1 and BLHP-B1 are illustrated in Table 1 accompanying a comparison with reported polysaccharide structures.

**TABLE 1:** The signal chemical shifts (ppm) of the major structures detected by NMR spectra of BLHP-A1 and BLHP-B1 samples.

Major units	Chemical shifts (δ_C_/δ_H_)/ppm						-CH_3_
	C1/H1	C2/H2	C3/H3	C4/H4	C5/H5	C6/H6	
**BLHP-A1**							
β-_D_-Man*p*-(1→	100.1/4.60	70.3/3.71	71.6/3.93	73.5/3.64	75.9/3.36	63.4/3.20	
α-_D_-Glc*p*-(1→	98.6/5.17	71.2/3.63	73.9/3.80	73.2/3.86	72.8/3.92	60.7/3.75	
β-_D_-Gal*p*-(1→	103.9/4.81	72.5/3.62	73.8/3.79	73.2/3.61	74.2/4.93	62.4/3.75[1	
→5)-β-_D_-Man*p*-(1→	101.1/4.52	76.3/3.90	76.3.0/3.70	76.8/3.37	82.5/4.15	69.7/3.30	
→3)-β-_D_-Gal*p*-(1→	101.9/4.41	72.7/3.55	78.1/4.04	68.2/3.91	75.6/3.72	65.3/3.70	
→4)-β-_D_-Xyl*p*-(1→	101.7/4.46	74.0/3.21	74.9/3.48	76.4/3.71	63.0/4.04		
→5)-α-_L_-Ara*f*-(1→ ^a^	105.1/5.23	81.6/4.06	78.2/3.62	85.9/3.97	66.8/3.67		
2-*O*-acetyl group							20.07/2.08
**BLHP-B1**							
β-_D_-Man*p*-(1→	100.1/4.60	70.3/3.71	71.6/3.93	73.5/3.64	75.9/3.36	63.4/3.20	
α-_D_-Glc*p*-(1→	98.6/5.18	71.2/3.65	73.8/3.77	73.2/3.82	72.7/3.90	60.7/3.71	
β-_D_-Gal*p*-(1→	103.9/4.81	72.5/3.62	73.8/3.79	73.2/3.61	74.2/4.93	62.4/3.75[1	
→5)-β-_D_-Man*p*-(1→	101.1/4.52	76.3/3.90	76.3.0/3.70	76.8/3.37	82.5/4.15	69.7/3.30	
→6)-α-_D_-Glc*p*-(1→	96.6/5.03	74.4/3.4	75.9/3.5	74.6/3.2	75.5/3.6	69.3/3.9[1	
→6)-β-_D_-Gal*p*-(1→	102.4/4.48	73.6/3.74	68.6/3.58	72.1/3.39	63.9/3.79	73.6/3.19	
→4)-β-_D_-Xyl*p*-(1→	101.7/4.42	73.7/3.25	74.9/3.52	76.4/3.73	67.0/3.38		
→5)-α-_L_-Ara*f*-(1→ ^a^	110.5/5.17	82.6/4.12	77.6/3.83	81.9/4.07	67.8/3.92		
2-*O*-acetyl group							20.01/2.10
3-*O*-acetyl group							22.30/2.18

*^a^ →5)-α-_L_-Araf-(1→ was determined by reported literature and NMR analysis while it was trace in methylation analysis.*

The 101.7/4.46 ppm was assigned to →4)-β-_D_-Xyl*p*-(1→ due to strong β-configuration at 4.46 ppm in BLHP-A1 (39). On the other hand, HPAEC analysis also indicated xylose was the dominant sugar in hydrolysate. The other ^1^H and ^13^C cross assignments for this structure were confirmed according to the HSQC spectrum. In addition, the ^1^H signals at about 2.1 ppm were attributed to *O*-acetyl groups linked as the terminal branches in the xylan skeleton (39). The trace and sporadic anomeric carbon signals at 109.1/5.32 ppm (in BLHP-A1) and 110.5/5.17 ppm (in BLHP-B1) in the HSQC spectrum (Figures 5C1,C2) were assigned to →5)-α-_L_-Ara*f*-(1→structure (40, 41). According to previous literature and NMR spectrum, the anomeric carbon signals near 102 ppm were attributed to C1 in Gal*p* relevant units (36, 42). The cross-peaks at 99.7/5.12 and 100.5/5.12 ppm were ascribed to δ_C1_/δ_H1_ of →4)-α-_D_-Glc*p*-(1→ units in BLHP-A1 and BLHP-B1, respectively, as well as 98.6/5.17 and 98.6/5.18 ppm, were determined as α-_D_-Glc*p*-(1→ (35, 43). Additionally, 59.7/3.36 ppm was attributed to OCH_3_ (44), which implied that monosaccharides residues in both of the fractions might be methylated naturally and partially.

### The Chemical Antioxidant Activities of Bamboo Leaves Hetero-Polysaccharides

**FIGURE 6:**
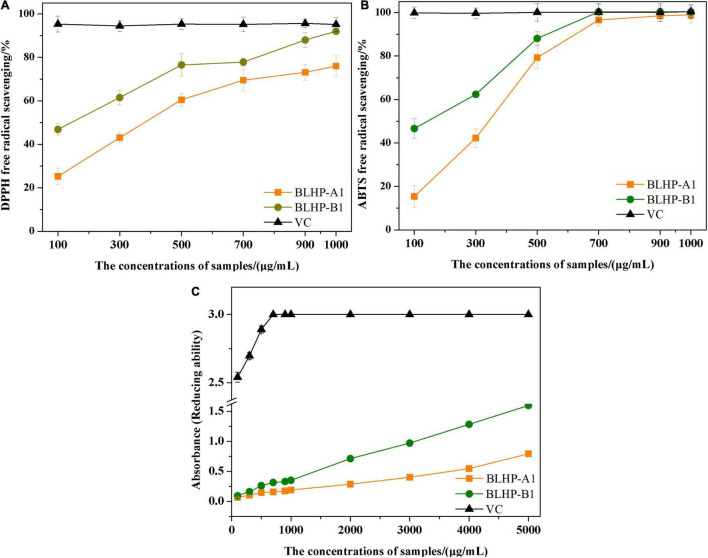
The measurements of antioxidant activities of BLHP-A1 and BLHP-B1 in chemical view, compared with relevant abilities of VC. **(A)** DPPH⋅ radicals scavenging of BLHP-A1 and BLHP-B1 samples in the concentration range of 100–1,000 μg/ml. **(B)** ABTS^+^ radicals scavenging of BLHP-A1 and BLHP-B1 samples in the concentration range of 0.1–1.0 mg/ml. **(C)** Reducing ability of BLHP-A1 and BLHP-B1 samples. The absorbances were recorded at 700 nm.

To evaluate the chemical antioxidant activity, DPPH⋅ and ABTS^+^ radicals scavenging activities and reducing ability were measured and the results are illustrated in Figure 6. In this work, the concentrations of BLHP samples from 100 to 1,000 μg/ml were employed to explore the DPPH⋅ and ABTS^+^ radical scavenging. As depicted in Figure 6A, both BLHP-A1 and BLHP-B1 samples showed the DPPH⋅ radical scavenging activity increased as the concentrations of BLHP rose while the same concentrations of vitamin C (VC) exhibited higher activity of radical scavenging. The scavenging rates of BLHP-A1 and BLHP-B1 for DPPH⋅ were 76.0 and 92.0%, respectively, when 1,000 μg/ml of samples were introduced. Hence, both samples demonstrated antioxidant activity for DPPH⋅ radicals scavenging. Moreover, the dosage-effect relationship was found between DPPH⋅ radical scavenging and the concentration of samples. IC_50_ of BLHP-A1 and BLHP-B1 were 330 and 140 μg/ml, respectively. According to previous studies, some polysaccharides extracted from plants or their appurtenances showed similar antioxidant activities but different intensities. Li’s group reported the polysaccharides from *Gynura procumbens* leaves acquired DPPH⋅ radical scavenging activity with 2,070 μg/ml for IC_50_ value (45). The polysaccharides from mung bean skin exhibited relatively high antioxidant activity, indicating a moderate effect on DPPH⋅ scavenging with the IC_50_ of 470 μg/ml (23).

ABTS^+^ radical scavenging activity of BLHP samples (Figure 6B) demonstrated a similar dosage-effect relationship. Unlike DPPH⋅ radical scavenging, the lower concentrations (300–700 μg/ml) of samples showed a relatively higher scavenging rate. When the concentration of BLHP was 700 μg/ml, ABTS^+^ radical scavenging rates of BLHP-A1 and BLHP-B1 were up to 96.6 and 100%, implying BLHP exhibited significant scavenging ability on ABTS^+^ in a concentration-dependent manner. IC_50_ of BLHP-A1 and BLHP-B1 for ABTS^+^ scavenging were 270 μg/ml and 130 μg/ml, respectively. A similar scavenging rate for ABTS^+^ by the polysaccharides from *Sagittaria sagittifolia* L needed 5,000 μg/ml which was significantly higher than that of BLHP (33). On the other hand, both DPPH⋅ and ABTS^+^ radical scavenging demonstrated that BLHP-B1 exhibited stronger activity than that of BLHP-A1. Probably, this promotion of the antioxidant activity was relevant to the linkage type in the structure of BLHP-B1. Moreover, high radical scavenging activities could be obtained as high concentrations of BLHP samples were employed (>700 μg/ml). From these views, the BLHP indicated moderate radical scavenging activities compared to positive control by concentrated VC.

The reducing ability was also regarded as a significant indicator of antioxidant activity. During the transformation of Fe^3+^ or ferricyanide complex to Fe^2+^, the electron-donating ability of polysaccharides could be occupied. The absorbance served as the indicator was measured at 700 nm (Figure 6C). The absorbances of BLHP-A1 and BLHP-B1 improved as the sample concentrations increased, while the reducing ability of BLHP was lower than that of VC at the same concentration.

To have a macroscopical knowledge of the antioxidant activity, we gave a comparison with recently reported polysaccharides in Table 2. The polysaccharides extracted from diverse sources showed various free radical scavenging, indicated by IC50 values of DPPH⋅ and ABTS^+^ radical scavenging. According to the published results, the polysaccharides in this work demonstrated relatively higher DPPH⋅ and ABTS^+^ radical scavenging, inspiring some potential applications in antioxidant materials manufacturing. Moreover, different extraction methods brought distinct molecular weights and chemical compositions which were regarded as one of the main factors to affect its functions. However, up to now, efficient extraction methods to produce polysaccharides with uniform molecular weight have not been well developed yet. But, it was certain that all reported active polysaccharides possessed heterogeneous monosaccharides constituted by abundant branches.

**TABLE 2:** A mini summary for DPPH⋅ and ABTS^+^ radical scavenging with molecular weight (MW) of some recent reported polysaccharides.

Polysaccharides source	Molar ratio of monosaccharides	MW/kDa	DPPH⋅ scavenging activity (IC50)/(mg/ml)	ABTS^+^ scavenging activity (IC50)/(mg/ml)	References
Mung bean skin	Rha^a^, Ara, Gal, Glc, Xyl, fructose (Fru), and GalA	208	0.47	0.37	(23)
*Auriculariales*	Man(85.0), GlcA(0.6), GalA(0.03), Glc(0.03), Gal(10.0), Xyl(0.1), Ara(3.6) and Fuc(0.6)	1260	1.0	None	(30)
*Sagittaria sagittifolia* L.^b^	_L_-Rha(8.47), _D_-Ara(2.09), _D_-Glu(75.01) and _D_-Gal(14.43)	1984.0	∼2.2	∼1.4	(33)
*Sagittaria sagittifolia* L.	_L_-Rha(1.24), _D_-Ara(0.22),_D_-Xly(0.49), _D_-Man(0.33), _D_-Glu(96.9) and _D_-Gal(0.81)	294.9	∼5.0^c^	∼4.5	(46)
*Holothuria leucospilota*	Rha(39.08), Fucose (Fuc, 35.72), GlcA(10.72), Gal(8.43), Glc(4.23) and Xly(1.83)	52.8	∼0.2	∼0.5	(47)
*Sargassum tenerrimum*	Fuc(52.3), Gal(1.7), Man(3.8), Ara(4.1)	31.18	∼0.1	> 0.125	(48)
*Plantago ovata Forssk* seeds	_L_-Rha(15.9), _D_-Xly(57.3), _L_-Ara(0.4), _D_-Glc(26.0) and _D_-Gal(0.4)	37.4	0.362	0.372	(49)
Oka	Gal(33.8–38.6), Glc(12.4–17.9), Rha(13.7–17.5), Ara(3.0–9.9) and GalA(19.0–21.2) and GlcA(6.7–8.3)	129	None	2.34	(50)
*Sargassum pallidum*	Fuc(14.93), Gal(26.63) and GalA(32.19)	510	∼0.5	∼0.5	(51)
Flaxseed hull	Glc, Gal, Xly and Ara	1696	∼0.5	∼0.31	(52)
Bamboo *(Phyllostachys pubescens Mazel)* leaves	_L_-Ara(1.00), _D_-Gal(2.44), _D_-Glu(0.18) and _D_-Xly(5.82)	8.53	0.14	0.13	*This work*

*^a^Molar ratio of monosaccharides was absent.*

*^b^Extracted by different methods.*

*^c^The IC50 value was estimated.*

### Bamboo Leaves Hetero-Polysaccharides Attenuation in Oxidative Stress Induced by H_2_O_2_ in HepG2 Cells

#### Effect of Bamboo Leaves Hetero-Polysaccharides and H_2_O_2_ on the Viability of HepG2 Cells

**FIGURE 7:**
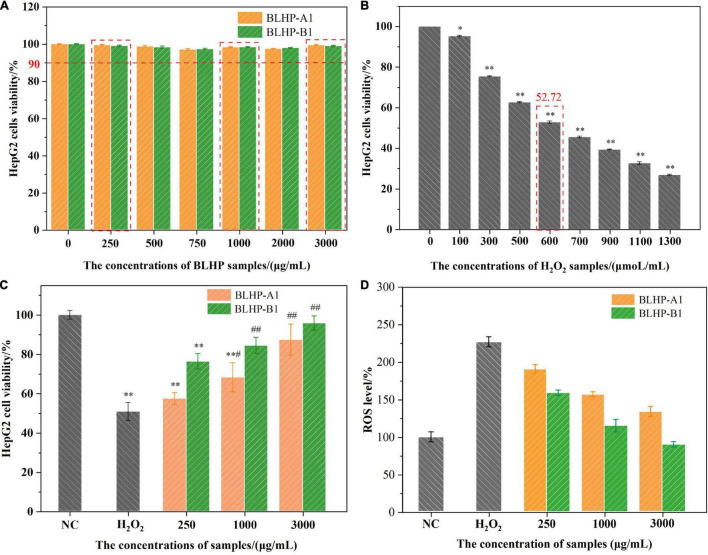
The effects of BLHP and H_2_O_2_ concentrations on HepG2 cells viability. **(A)** The HepG2 cells were incubated with BLHP samples at different concentrations (0, 250, 500, 750, 1,000, 2,000, and 3,000 μg/ml). **(B)** The HepG2 cells were treated with H_2_O_2_ at different concentrations (0, 100, 300, 500, 600, 700, 900, 1,100, and 1,300 μmol/ml). **(C)** The treated concentrations of H_2_O_2_, BLHP samples in the oxidative stress model. **(D)** Variations in relative ROS levels compared to that of NC group. The selected concentration of H_2_O_2_ was 600 μmoL/ml. The selected concentrations of BLHP samples were 250, 1,000 and 3,000 μg/ml. (*p < 0.05, ^**^p < 0.01 compared with the control group; #*p* < 0.05, ##*p* < 0.01 compared with the H_2_O_2_ group).

The cytotoxicity of HepG2 cells affected by different concentrations of polysaccharides and H_2_O_2_ solutions was evaluated by the MTT method and the results are illustrated in Figure 7. Fortunately, the viability of the cells in BLHP solutions was more than 90% and near 98% in the concentration range of 250–3,000 μg/ml (Figure 7A), implying the toxicities of BLHP-A1 and BLHP-B1 were exceedingly weak. Comparatively, H_2_O_2_-induced cells exhibited apoptosis intensively depending on the H_2_O_2_ concentration from 100 to 1,300 μmoL/ml (Figure 7B). To be noted, 600 μmoL/ml of H_2_O_2_ could result in 52.72% of cell viability, close to half of the cell viability. These results inferred that the model of H_2_O_2–_induced HepG2 cells was established employing 600 μmoL/ml of H_2_O_2_. Moreover, 250, 1,000, and 3,000 μg/ml of BLHP samples should be introduced on behalf of low, medium, and high dosages as concluded in Figure 7C. What was worth mentioning was the practically 100% viability of HepG2 cells in the negative control (NC) group. Furthermore, H_2_O_2_ caused the production of larger quantities of ROS in HepG2 cells, indicating 2.5-folds higher than that in non-induced cells (Figure 7D). The addition of BLHP samples inhibited the relative ROS level in H_2_O_2_-induced cells, implying BLHP possessed a potential protective function for oxidative damage.

#### H_2_O_2_-Induced Oxidative Damage of HepG2 Cells

**FIGURE 8:**
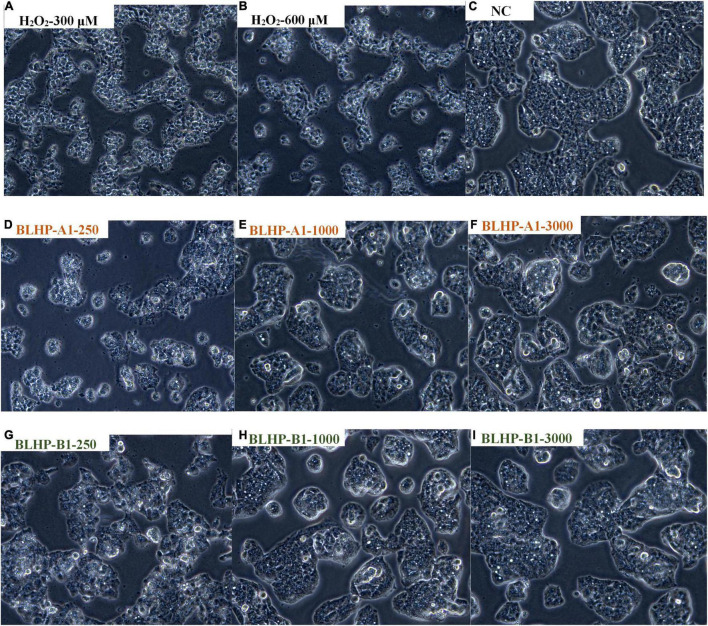
The morphologies of HepG2 cells in different situations. **(A,B)** Were treated with 300 and 600 μmol/ml H_2_O_2_, respectively. **(C)** Represented natural cells in MEM without any treatment or protection. **(D–F)** Illustrated the protection effects in H_2_O_2_-induced cells by various concentrations (250, 1,000, and 3,000 μg/ml) of BLHP-A1. **(G–I)** Indicated the protection effects in H_2_O_2_-induced cells by various concentrations (250, 1,000, and 3,000 μg/ml) of BLHP-B1.

The growth morphologies of HepG2 cells in positive, negative, and induced groups exhibited conspicuous differences. The cell counts in induced groups (Figures 8A,B) reduced significantly compared with the NC group (Figure 8C), suggesting the oxidative damage from H_2_O_2_ suppressed the cell viability. Notably, the normal HepG2 cells were epithelial-like growing as monolayers (adherent growth) in small aggregates. From the cell morphologies in positive groups (Figures 8D–I), we could easily conclude that BLHP was rewarding for protection against oxidative damage. Merely from the number of morphological mutants, BLHP-B1 seemingly possessed a more substantial protective effect than that of BLHP-A1 on oxidative damage, especially in low dosage (250 μg/ml). More adhered cells were observed in the BLHP-B1-treated group. Furthermore, with the increase of BLHP concentrations, the growing shapes of HepG2 cells dramatically changed along with natural orientation nearly in a concentration-dependent manner. The cells emerged as typical morphologies on the verge of that in the NC group as the concentration of BLHP-B1 was 3,000 μg/ml.

#### Protective Measurement of Bamboo Leaves Hetero-Polysaccharides on H_2_O_2_-Induced Oxidative Damage in HepG2 Cells

**FIGURE 9:**
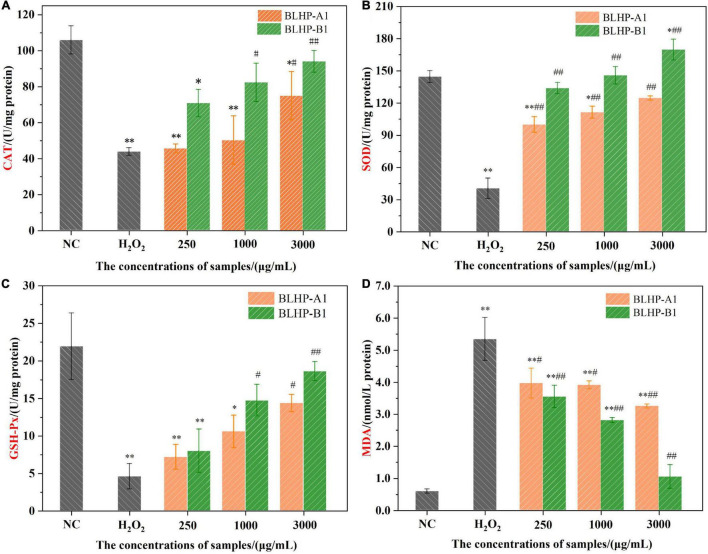
The effects of BLHP samples on the concentrations of antioxidant enzymes CAT **(A)**, SOD **(B)**, GSH-Px **(C)** and MDA **(D)** product. NC, normal control; H_2_O_2_: H_2_O_2_ (600 μmoL/ml) induced group. (*p < 0.05, ^**^p < 0.01 compared with the control group; #*p* < 0.05, ##*p* < 0.01 compared with the H_2_O_2_ group).

According to the chemical antioxidant activities and the morphologies of HepG2 cells in positive groups, we might harbor the idea that BLHP, especially BLHP-B1, demonstrated the promising ability to scavenge free radicals and prosperity of cell viability. These results captured our attention to further explore the protective effect on H_2_O_2_-induced oxidative damage in HepG2 cells. Hence, some indicators, including the activities of SOD, CAT, and GSH-Px enzymes, MDA products in oxidative stress, were evaluated and measured. Many polysaccharides could enhance enzyme activity or restore the antioxidant enzymes system to scavenge the accumulating free radicals in the body for retarding aging (53, 54). H_2_O_2_ was a quintessential inductive agent which could result in the accumulation of free radicals and damage the antioxidant enzymes (55, 56). In this study, we established H_2_O_2_-induced damaged HepG2 cells to investigate the protective effect of BLHP on oxidative stress. Figure 9 illustrates the activities of CAT, SOD, and GSH-Px in H_2_O_2_-treated groups. In detail, the activities of CAT, SOD, and GSH-Px were decreased to 41.3, 27.8, and 20.8%, respectively, compared to that in the negative control group as illustrated in Figures 9A–C. Interestingly, the BLHP-treated groups overwhelmingly gave rise to the activities of CAT, SOD, and GSH-Px in H_2_O_2_-treated cells, even reverting or exceeding them to baselines. The activity of the SOD enzyme compared to that in the control group was even slightly enhanced when 3,000 μg/ml of BLHP-B1 was employed (Figure 9B). Furthermore, there was another phenomenon that fascinated us. BLHP-B1 sample expressed stronger protection even promoting the ability to the activities of CAT, SOD, and GSH-Px than those of BLHP-A1. Hence, the different structure fragments including →6)-β-_D_-Gal*p*-(1→ and 3-*O*-acetyl group, might play an essential role in the attenuation of oxidative stress in HepG2 cells. Probably, the advanced structure of polysaccharides played a greater role in determining their bioactivities. However, we had enough advanced technologies to parse the secondary or even tertiary structure of these polysaccharides. Moreover, the enhancements for the enzyme system were all performed in a dosage-dependent manner in low, medium, and high concentrations of BLHP. A significant increase to 5.34 nmol/L of the MDA previously exposed to 600 μmol/ml H_2_O_2_ for 4.0 h (Figure 9D) while the negative control group showed trace concentrations of MDA. The MDA content in the BLHP-treated groups was extremely decreased coinciding with the dosage effect. These findings elucidated that the H_2_O_2_-injured cells were remarkably attenuated after treatment with 250, 1,000, and 3,000 μg/ml of BLHP.

Some endogenous antioxidant enzymes including catalase (CAT), SOD, and GSH-Px constituted a self-defense system against oxidative stress (57). The BLHP treatment increased the CAT activity, implying the cells possessed the capacity to purge H_2_O_2_ conversion directly to oxygen and water while H_2_O_2_ decreased the activity of the CAT enzyme. During the H_2_O_2_ treatment, some free radicals would release successively resulting in the accumulation of oxidizing species. Likewise, the SOD enzyme activity reflected the loading of intracellular antioxidants, further indicating the intracellular radical’s levels. BLHP acted as a promoter of SOD activity besides scavenging free radicals as proved in Figure 9B. GSH-Px performed as one of the essential components in the antioxidant defense system, serving as an electron donor to insecure ROS. The metabolism of GSH was deduced to be regulated by bamboo leaves polysaccharides to avoid cell apoptosis. Previous studies of oxidative stress induced by H_2_O_2_ had emphasized that the caspase enzyme system could be inhibited by polysaccharides (58, 59). Besides, the expression of other proteins promoting the apoptosis process would be coincidentally inhibited by polysaccharides.

## Conclusion

In this study, we extracted hetero-polysaccharides (BLHP) from bamboo leaves assisted by a small amount of phosphotungstic acid. Chemical and physical characterizations proved that BLHP-A1 and BLHP-B1 fractions belonged to hetero-polysaccharides. Successively, we evaluated the antioxidant activity of BLHP. As a result, both the fractions demonstrated the potential to scavenge DPPH⋅ and ABTS^+^ radicals and exhibited relatively high reducing ability in *vitro* antioxidant assays. Furthermore, BLHP-B1 showed more activity to prevent H_2_O_2_-induced damages in HepG2 cells. BLHP-B1 could suppress oxidant stress by improving the enzyme activities of SOD, CAT, and GSH-Px as well as decreasing the production of MDA. According to the morphologies of damaged HepG2 cells and the enzyme activities, it was proved that BLHP, especially BLHP-B1, could attenuate injury in oxidative stress in HepG2 cells. These results hinted that hemicellulosic polysaccharides extracted by heteropolyacid from bamboo leaves possessed the potential to be used as an antioxidant for the organism but further *in vivo* and mechanism studies need lucubrating.

## Data Availability Statement

The raw data supporting the conclusions of this article will be made available by the authors, without undue reservation.

## Author Contributions

ZX contributed to the conceptualization, methodology, and writing of the original draft. JL and QZ performed the investigation and collected resources. HW conducted a formal analysis. QG used software for analysis and validation. JM and RS contributed to the project administration and supervision. All authors contributed to the article and approved the submitted version.

## Conflict of Interest

The authors declare that the research was conducted in the absence of any commercial or financial relationships that could be construed as a potential conflict of interest.

## Publisher’s Note

All claims expressed in this article are solely those of the authors and do not necessarily represent those of their affiliated organizations, or those of the publisher, the editors and the reviewers. Any product that may be evaluated in this article, or claim that may be made by its manufacturer, is not guaranteed or endorsed by the publisher.

## References

[1] Yu S Liu GC Wang ML Lv ZY Du PG. A selenium polysaccharide from Platycodon grandiflorum rescues PC12 cell death caused by H2O2 via inhibiting oxidative stress. *Int J Biol Macromol.* (2017) 144:393–9. 10.1016/j.ijbiomac.2017.06.052 28610929

[2] Duračková Z. Some current insight into oxidative stress. *Physiol Res.* (2009) 59:459–69. 10.33549/physiolres.931844 19929132

[3] Bhat AH Dar KB Anees S Zargar MA Masood A Sofi MA Oxidative stress, mitochondrial dysfunction and neurodegenerative diseases; a mechanistic insight. *Biomed Pharmacother.* (2015) 74:101–10. 10.1016/j.biopha.2015.07.025 26349970

[4] Li Y Sun Y Zhu MZ Zhu RX Zhang JZ Zhou JC Sacculatane diterpenoids from the Chinese liverwort *Pellia epiphylla* with protection against H2O2-induced apoptosis of PC12 cells. *Phytochemistry.* (2019) 162:173–82. 10.1016/j.phytochem.2019.03.007 30925378

[5] Li XX Rommelaere S Kondo S Lemaitre B. Renal purge of hemolymphatic lipidsprevents the accumulation of ROS-induced inflammatory oxidized lipids and protects drosophila from tissue damage. *Immunity.* (2020) 52:374–87.e6. 10.1016/j.immuni.2020.01.008 32075729

[6] Wong FC Xiao JB Wang SY Ee KY Chai TT. Advances on the antioxidant peptides from edible plant sources. *Trends Food Sci Technol.* (2020) 99:44–57. 10.1016/j.tifs.2020.02.012

[7] Li TT Wu CE Meng XY Fan GJ Tang Y. Structural characterization and antioxidant activity of a glycoprotein isolated from *Camellia oleifera* Abel seeds against D-galactose-induced oxidative stress in mice. *J Funct Foods.* (2020) 64:e103594. 10.1016/j.jff.2019.103594

[8] Ji XL Cheng YQ Tian JY Zhang SQ Jing YS Shi MM. Structural characterization of polysaccharide from jujube (*Ziziphus jujuba* Mill.) fruit. *Chem Biol Technol Agric.* (2021) 8:54–60. 10.1186/s40538-021-00255-2

[9] Szpakowska N Kowalczyk A Kaczynski Z. The chemical structure of polysaccharides isolated from the *Ochrobactrum rhizosphaerae* PR17T. *Carbohydr Res.* (2020) 497:e108136. 10.1016/j.carres.2020.108136 32889436

[10] Ferreira I Heleno SA Reis FS Stojkovic D Queiroz MJ Vasconcelos MH Chemical features of *Ganoderma polysaccharides* with antioxidant, antitumor and antimicrobial activities. *Phytochemistry.* (2015) 114:38–55. 10.1016/j.phytochem.2014.10.011 25457487

[11] Peng H Wang N Hu ZR Yu ZP Liu YH Zhang JS Physicochemical characterization of hemicelluloses from bamboo (*Phyllostachys pubescens* Mazel) stem. *Ind Crop Prod.* (2012) 37:41–50.

[12] Peng P She D. Isolation, structural characterization, and potential applications of hemicelluloses from bamboo: a review. *Carbohyd Polym.* (2014) 112:701–20. 10.1016/j.carbpol.2014.06.068 25129800

[13] Gilbert HJ Knox JP Boraston AB. Advances in understanding the molecular basis of plant cell wall polysaccharide recognition by carbohydrate-binding module. *Curr Opin Struc Biol.* (2013) 23:669–77. 10.1016/j.sbi.2013.05.005 23769966

[14] Ji XL Guo JH Pan FB Kuang FJ Chen HM Guo XD Structural elucidation and antioxidant activities of a neutral polysaccharide from Arecanut (*Areca catechu* L.). *Front Nutr.* (2022) 9:e853115. 10.3389/fnut.2022.853115 35340550 PMC8948432

[15] Mirzadeh M Arianejad MR Khedmat L. Antioxidant, antiradical, and antimicrobial activities of polysaccharides obtained by microwave-assisted extraction method: a review. *Carbohyd Polym.* (2020) 229:e115421. 10.1016/j.carbpol.2019.115421 31826454

[16] Surayot US Yelithao K Tabarsa M Lee DH Palanisamy S Prabhu NM Structural characterization of a polysaccharide from *Certaria islandica* and assessment of immunostimulatory activity. *Process Biochem.* (2019) 83:214–21. 10.1016/j.procbio.2019.05.022

[17] Xiao ZQ Wang XL Yang QQ Xing C Ge Q Gai XK Ball milling promotes saccharification of agricultural biomass by heteropolyacid and enzyme: unlock the lignin cage for sugars recovery. *Biomass Convers Biorefin.* (2020) 9:12–22. 10.1007/s13399-020-00950-4

[18] Ji XL Guo JH Ding DQ Gao J Hao LR Guo XD Structural characterization and antioxidant activity of a novel high-molecular-weight polysaccharide from *Ziziphus Jujuba* cv. Muzao. *J Food Meas Charact.* (2022) 2:e1288. 10.1007/s11694-022-01288-3

[19] Li WL Lin K Zhou M Xiong Q Li CY Ru Q. Polysaccharides from *Opuntia milpa* alta alleviate alloxan-induced INS-1 cells apoptosis via reducing oxidative stress and upregulating Nrf2 expression. *Nutr Res.* (2020) 77:108–18. 10.1016/j.nutres.2020.02.004 32422500

[20] Wen ZS Xue R Du M Tang Z Xiang XW Zheng B Hemp seed polysaccharides protect intestinal epithelial cells from hydrogen peroxide-induced oxidative stress. *Int J Biol Macromol.* (2019) 135:203–11. 10.1016/j.ijbiomac.2019.05.082 31108145

[21] Jeong HK Lee D Kim HP Baek SH. Structure analysis and antioxidant activities of an amylopectin-type polysaccharide isolated from dried fruits of *Terminalia chebula*. *Carbohyd Polym.* (2019) 211:100–8. 10.1016/j.carbpol.2019.01.097 30824068

[22] Chaiklahana R Chirasuwana N Triratanaa P Lohab V Tiab S Bunnag B. Polysaccharide extraction from *Spirulina* sp. and its antioxidant capacity. *Int J Biol Macromol.* (2013) 58:73–8. 10.1016/j.ijbiomac.2013.03.046 23541559

[23] Jiang L Wang WJ Wen PW Shen MY Li HR Ren YM Two water-soluble polysaccharides from mung bean skin: physicochemical characterization, antioxidant and antibacterial activities. *Food Hydrocoll.* (2020) 100:e105412. 10.1016/j.foodhyd.2019.105412

[24] Ma JS Liu H Han CR Zeng SJ Xu XJ Lu DJ Extraction, characterization and antioxidant activity of polysaccharide from *Pouteria campechiana* seed. *Carbohyd Polym.* (2020) 229:e115409. 10.1016/j.carbpol.2019.115409 31826479

[25] Xiao ZQ Zhang Q Dai J Wang XL Yang QQ Cai CG Structural characterization, antioxidant and antimicrobial activity of water-soluble polysaccharides from bamboo (*Phyllostachys pubescens* Mazel) leaves. *Int J Biol Macromol.* (2020) 142:432–42. 10.1016/j.ijbiomac.2019.09.115 31593720

[26] Chale-Dzul J Freile-Pelegrín Y Robeldo D Moo-Puc R. Protective effect of fucoidans from tropical seaweeds against oxidative stress in HepG2 cells. *J Appl Phycol.* (2017) 29:2229–38. 10.1007/s10811-017-1194-3

[27] Guo QW Xu LL Chen Y Ma QQ Santhanam RK Xue ZH Structural characterization of corn silk polysaccharides and its effect in H2O2 induced oxidative damage in L6 skeletal muscle cells. *Carbohyd Polym.* (2019) 208:161–7. 10.1016/j.carbpol.2018.12.049 30658787

[28] Gao CP Zhong LF Jiang LP Geng CY Yao XF Cao J. *Phellinus linteus* mushroom protects against tacrine-induced mitochondrial impairment and oxidative stress in HepG2 cells. *Phytomedicine.* (2013) 20:705–9. 10.1016/j.phymed.2013.02.014 23523257

[29] Mehmood T Maryam A Zhang H Li YM Khan M Ma TH. Deoxyelephantopin induces apoptosis in HepG2 cells via oxidative stress, NF-κB inhibition and mitochondrial dysfunction. *Biofactors.* (2016) 43:63–72. 10.1002/biof.1324 27628030

[30] Su Y Li L. Structural characterization and antioxidant activity of polysaccharide from four auriculariales. *Carbohyd Polym.* (2020) 229:e115407. 10.1016/j.carbpol.2019.115407 31826485

[31] Peng H Zhou MY Yu ZP Zhang JS Ruan R Wan YQ Fractionation and characterization of hemicelluloses from young bamboo (*Phyllostachys pubescens* Mazel) leaves. *Carbohyd Polym.* (2013) 95:262–71. 10.1016/j.carbpol.2013.03.007 23618268

[32] Tang W Liu CC Liu JJ Hu LY Huang YS Yuan L Purification of polysaccharide from *Lentinus edodes* water extract by membrane separation and its chemical composition and structure characterization. *Food Hydrocoll.* (2020) 105:e105851. 10.1016/j.foodhyd.2020.105851

[33] Gu JY Zhang HH Zhang JX Wen CT Ma HL Duan YQ Preparation, characterization and bioactivity of polysaccharide fractions from *Sagittaria sagittifolia* L. *Carbohyd Polym.* (2020) 229:e115355. 10.1016/j.carbpol.2019.115355 31826432

[34] Zhu JX Chen ZY Chen L Yu C Wang HX Wei XL Comparison and structural characterization of polysaccharides from natural and artificial Se-enriched green tea. *Int J Biol Macromol.* (2019) 130:388–98. 10.1016/j.ijbiomac.2019.02.102 30794901

[35] Cao JJ Lv QQ Zhang B Chen HQ. Structural characterization and hepatoprotective activities of polysaccharides from the leaves of *Toona sinensis* (A. Juss) Roem. *Carbohyd Polym.* (2019) 212:89–101. 10.1016/j.carbpol.2019.02.031 30832884

[36] Chaves PFP Iacomini M Cordeiro LMC. Chemical characterization of fructooligosaccharides, inulin and structurally diverse polysaccharides from chamomile tea. *Carbohyd Polym.* (2019) 214:269–75. 10.1016/j.carbpol.2019.03.050 30925997

[37] Khemakhem I Abdelhedi O Trigui I Ayadi MA Bouaziz M. Structural, antioxidant and antibacterial activities of polysaccharides extracted from olive leaves. *Int J Biol Macromol.* (2018) 106:425–32. 10.1016/j.ijbiomac.2017.08.037 28802847

[38] Gong P Wang SY Liu M Chen FX Yang WJ Chang XN Extraction methods, chemical characterizations and biological activities of mushroom polysaccharides: a mini-review. *Carbohydr Res.* (2020) 494:e108037. 10.1016/j.carres.2020.108037 32592937

[39] Wang KL Wang B Hu RB Zhao XH Li HL Zhou GK Characterization of hemicelluloses in *Phyllostachys edulis* (moso bamboo) culm during xylogenesis. *Carbohyd Polym.* (2019) 221:127–36. 10.1016/j.carbpol.2019.05.088 31227151

[40] Zelaya VM Fernández PV Vega AS Mantese AI Federico AA Ciancia M. Glucuronoarabinoxylans as major cell walls polymers from youngshoots of the woody bamboo *Phyllostachys aurea*. *Carbohyd Polym.* (2017) 167:240–9. 10.1016/j.carbpol.2017.03.015 28433159

[41] Li C Dong ZP Zhang B Huang Q Liu G Fu X. Structural characterization and immune enhancement activity of a novel polysaccharide from *Moringa oleifera* leaves. *Carbohyd Polym.* (2020) 234:e115897. 10.1016/j.carbpol.2020.115897 32070517

[42] Seyfi R Kasaai MR Chaichi MJ. Isolation and structural characterization of a polysaccharide derived from a local gum: zedo (*Amygdalus scoparia* Spach). *Food Hydrocolloid.* (2019) 87:915–24. 10.1016/j.foodhyd.2018.09.017

[43] Shi WT Zhong J Zhang Q Yan CY. Structural characterization and antineuroinflammatory activity of a novel heteropolysaccharide obtained from the fruits of *Alpinia oxyphylla*. *Carbohyd Polym.* (2020) 229:e115405. 10.1016/j.carbpol.2019.115405 31826414

[44] Lin YP An FP He H Feng F Song HB Huang Q. Structural and rheological characterization of pectin from passion fruit (*Passiflora edulis* f. flavicarpa) peel extracted by high-speed shearing. *Food Hydrocoll.* (2021) 114:e106555. 10.1016/j.foodhyd.2020.106555

[45] Li C Li X You L Fu X Liu RH. Fractionation, preliminary structural characterization and bioactivities of polysaccharides from *Sargassum pallidum*. *Carbohyd Polym.* (2017) 155:261–70. 10.1016/j.carbpol.2016.08.075 27702511

[46] Gu JY Zhang HH Yao H Zhou J Duan YQ Ma HL. Comparison of characterization, antioxidant and immunological activities of three polysaccharides from *Sagittaria sagittifolia* L. *Carbohyd Polym.* (2019) 133:11–20. 10.1016/j.carbpol.2020.115939 32122481

[47] Yuan YQ Li C Zheng QW Wu JX Zhu KX Shen XR Effect of simulated gastrointestinal digestion in vitro on the antioxidant activity, molecular weight and microstructure of polysaccharides from a tropical sea cucumber (*Holothuria leucospilota*). *Food Hydrocoll.* (2019) 89:735–41. 10.1016/j.foodhyd.2018.11.040

[48] Raguraman V Abraham LS Jyotsna J Seedevi P Kannan GS Thirugnanasambandam R Sulfated polysaccharide from *Sargassum tenerrimum* attenuates oxidative stress induced reactive oxygen species production in in vitro and in zebrafish model. *Carbohyd Polym.* (2019) 203:441–9. 10.1016/j.carbpol.2018.09.056 30318233

[49] Patel MK Tanna B Gupta H Mishra A Jha B. Physicochemical, scavenging and anti-proliferative analyses of polysaccharides extracted from psyllium (*Plantago ovata* Forssk) husk and seeds. *Int J Biol Macromol.* (2019) 133:190–201. 10.1016/j.ijbiomac.2019.04.062 30981777

[50] Olawuyi IF Kim SR Hahn D Lee WY. Influences of combined enzyme-ultrasonic extraction on the physicochemical characteristics and properties of okra polysaccharides. *Food Hydrocoll.* (2020) 100:e105396. 10.1016/j.foodhyd.2019.105396

[51] Yuan D Li C Huang Q Fu X. Ultrasonic degradation effects on the physicochemical, rheological and antioxidant properties of polysaccharide from *Sargassum pallidum*. *Carbohyd Polym.* (2020) 239:e116230. 10.1016/j.carbpol.2020.116230 32414439

[52] Yuan B Han JN Cheng YL Cheng SJ Huang DC Mcclements DJ Identification and characterization of antioxidant and immune-stimulatory polysaccharides in flaxseed hull. *Food Chem.* (2020) 315:e126226. 10.1016/j.foodchem.2020.126266 32000083

[53] Yan L Xiong C Zhu P Xu J Yang ZR Ren H Structural characterization and in vitro antitumor activity of a polysaccharide from *Artemisia annua* L. (Huang Huahao). *Carbohyd Polym.* (2019) 213:361–9. 10.1016/j.carbpol.2019.02.081 30879680

[54] Kumar PP Prashanth KVH. Low molecular weight chitosan (~20 kDa) protects acrylamide induced oxidative stress in D. melanogaster by restoring dopamine and KIF5B levels. *Carbohyd Polym.* (2019) 222:e115005. 10.1016/j.carbpol.2019.115005 31320041

[55] Trinh MDL Ngo DH Tran DK Tran QT Vo TS Dinh MH Prevention of H2O2-induced oxidative stress in Chang liver cells by 4-hydroxybenzyl-chitooligomers. *Carbohyd Polym.* (2014) 103:502–9. 10.1016/j.carbpol.2013.12.061 24528760

[56] Chinnapaka S Zheng GX Chen AS Munirathinam G. Nitro aspirin (NCX4040) induces apoptosis in PC3 metastatic prostate cancer cells via hydrogen peroxide (H2O2)-mediated oxidative stress. *Free Radic Biol Med.* (2019) 143:494–509. 10.1016/j.freeradbiomed.2019.08.025 31446057 PMC6848783

[57] Skalski B Lis B Pecio Ł Kontek B Olas B Żuchowski J Isorhamnetin and its new derivatives isolated from sea buckthorn berries prevent H2O2/Fe–induced oxidative stress and changes in hemostasis. *Food Chem Toxicol.* (2019) 125:614–20. 10.1016/j.fct.2019.02.014 30738133

[58] Xiong C Li Q Chen C Chen ZQ Huang WL. Neuroprotective effect of crude polysaccharide isolated from the fruiting bodies of *Morchella importuna* against H2O2-induced PC12 cell cytotoxicity by reducing oxidative stress. *Biomed Pharmacother.* (2016) 83:569–76. 10.1016/j.biopha.2016.07.016 27459112

[59] Oh SH Vo TS Ngo DH Kim SY Ngo DN Kim SK. Prevention of H2O2-induced oxidative stress in murine microglial BV-2 cells by chitin-oligomers. *Process Biochem.* (2016) 51:2170–5. 10.1016/j.procbio.2016.08.015

